# Pistachio Consumption Prevents and Improves Lipid Dysmetabolism by Reducing the Lipid Metabolizing Gene Expression in Diet-Induced Obese Mice

**DOI:** 10.3390/nu10121857

**Published:** 2018-12-01

**Authors:** Simona Terzo, Gaetano Felice Caldara, Vincenzo Ferrantelli, Roberto Puleio, Giovanni Cassata, Flavia Mulè, Antonella Amato

**Affiliations:** 1 Dipartimento di Scienze e Tecnologie Biologiche, Chimiche e Farmaceutiche (STEBICEF), Università di Palermo, viale delle Scienze, Edificio 16, 90128 Palermo, Italy; simona.terzo01@unipa.it (S.T.); gaetanofelice.caldara@unipa.it (G.F.C); flavia.mule@unipa.it (F.M.); 2 Istituto Zooprofilattico Sperimentale della Sicilia “A. Mirri”, Via Gino Marinuzzi 3, 90129 Palermo, Italy; vincenzo.ferrantelli@izssicilia.it (V.F.); roberto.puleioizs@gmail.com (R.P.); giovanni.cassata@izssicilia.it (G.C.)

**Keywords:** pistachio consumption, obesity-related dysfunctions, lipid metabolizing gene expression

## Abstract

Pistachios contain beneficial substances such as unsaturated fatty acids, phytosterols, and polyphenols. In the present study, we investigated if pistachio consumption is able to prevent or to revert hyperglycemia, dyslipidemia, hepatic steatosis, and adipose tissue morphological alterations caused by high fat diet (HFD) in the mouse. Moreover, the impact of pistachio intake on the mRNA expression of peroxisome proliferator-activated receptor γ (*PPAR-γ*), fatty acid transport proteins (*FAT-P*), fatty acid synthase (*FAS*), stearoyl-CoA desaturase (*SCD1*), and sterol regulatory element-binding transcription factor-1c (*SREBP-1c*) in liver and adipose tissue was also analyzed. No change in body weight, food intake, and hyperglycemia was observed between mice consuming pistachios (HFD-P) and HFD mice. Pistachio intake was able to prevent but not to reverse HFD-induced hypertriglyceridemia. Cholesterol plasma levels, steatosis grading, body fat mass, and adipocyte size were significantly lower in HFD-P group compared to HFD in both prevention and reversal protocol. Pistachio-diet was able to prevent HFD-induced overexpression of *PPAR-γ*, *FAS*, and *SCD1* in the liver and *SREBP-1c, PPAR-γ*, and *FAT-P* in adipose tissue. Similarly, HFD-P significantly ameliorated the expression levels of *FAT-P* and *SCD1* in the liver and *SREBP-1c, FAS*, and *SCD1* in adipose tissue of obese mice. The present study shows that pistachio consumption is able to prevent and to ameliorate obesity-related dysfunctions by positively modulating the expression of genes linked to lipid metabolism.

## 1. Introduction

Metabolic syndrome (MetS) is a cluster of conditions that increase the risk of cardiovascular diseases and diabetes mellitus because it is characterized by obesity, hyperglycemia, insulin resistance, hypertension, and dyslipidemia. The pathogenesis of MetS is a complex issue involving genetic, environmental, and dietary factors [1]. It is well known that a high-fat-diet (HFD) and excessive nutrient intake result not only in adipose tissue (AT) triglyceride accumulation, with consequent adipocyte hypertrophy and pro-inflammatory cytokines release, but also in ectopic fat deposition. Excessive hepatic lipids can lead to steatosis, the initial stage of non-alcoholic fatty liver disease (NAFLD). NAFLD is strictly linked to atherogenic dyslipidemia and diabetes and it is considered to be a hepatic component of MetS [2]. Several metabolic and signaling pathways are involved in perpetrating the obesity-metabolic disturbances observed in the liver and AT. In particular, recent studies reported that hepatic or adipose tissue genes concerning synthesis and transport of fatty acids, including fatty acid transport proteins *(FAT-P*), sterol regulatory element-binding transcription factor-1c (*SREBP-1c*), stearoyl-CoA desaturase (*SCD1*), fatty acid synthase (*FAS*) and peroxisome proliferator-activated receptor γ (*PPAR-γ*), are upregulated in HFD rodents [3,4].

Diet-based strategies, such as foods with ipolipidemic, anti-oxidant, and anti-inflammatory properties, are recommended when dealing with MetS. 

Natural remedies are currently drawing attention as therapeutic or protective agents in treating MetS because natural plant compounds seem to improve obesity-related dysfunctions [5,6]. 

Health benefits of regular nut consumption (mainly pistachios, almonds, and walnuts) have been well-documented in studies both on animals and humans [7,8,9]. Daily nut consumption can improve dysmetabolic conditions such as obesity, type 2 diabetes mellitus (T2DM), and related cardiovascular diseases [10,11,12]. 

Compared to other nuts, pistachios have a higher amount of monounsaturated fatty acid (MUFA) and polyunsaturated fatty acid (PUFA) [9,13,14]. They are rich in phytosterols (stigmasterol and campesterol), lutein (xanthophyll carotenoid), and polyphenols (resveratrol and catechins) [9,10,11,12,13,14,15]. These substances are known for their anti-inflammatory and antioxidant actions [9,16,17,18]. Therefore, regular pistachio intake could have higher beneficial potential than other nuts. Although pistachios are commonly considered a fattening food, on account of their high fat content, different studies have shown that the addition of pistachios to ordinary diets did not induce weight gain [7,9,19]. The advantageous effects of regular pistachio intake on lipid profile remain controversial. In fact, previous studies on humans and animals reported decreases or no effect on low-density lipoprotein (LDL) after pistachio consumption [11,12,19,20,21,22,23,24,25].

Nevertheless, the potential beneficial properties of pistachio consumption on other obesity-related dysfunctions, such as hepatic steatosis and adipose tissue morphological alterations, have not been explored yet.

The purpose of the present study was to investigate if pistachio consumption is able to prevent or to revert obesity-related metabolic dysfunctions, such as hyperglycemia, dyslipidemia, hepatic steatosis, and AT morphological alterations, in HFD mice. In addition, the effects of regular pistachio intake on the expression of genes linked to fatty acid synthesis and lipid uptake, such as *SREBP-1c*, *PPAR-γ*, *SCD1*, *FAS*, and *FAT-P*, were analyzed in liver and adipose tissue to explore the mechanism of action responsible for the beneficial effects. 

## 2. Materials and Methods 

### 2.1. Animals

The procedures were performed in accordance with the Italian legislative decree No 26/2014, and the European directive 2010/63/UE.

The experimental protocols were approved by the animal welfare committee of the Istituto Zooprofilattico Sperimentale della Sicilia “A. Mirri” (Palermo, Italy) and authorized by the Ministry of Health (Rome, Italy; Authorization Number 349/2016-PR).

Four-week old male C57BL/6J (B6) mice, purchased from Harlan Laboratories (San Pietro al Natisone-Udine, Italy) were housed under standard conditions of light (12 h light:12 h darkness cycle) and temperature (22–24 °C), with free access to water and food. Mice were allowed to acclimate for 1 week prior to the implementation of the special diets. 

### 2.2. Prevention Study

Mice were randomly divided into three groups: (1) Lean group: control animals fed with standard diet (STD; 70% of energy as carbohydrates, 20% protein, and 10% fat; 4RF25 Mucedola, Milan, Italy) for 16 weeks; (2) High-fat diet (HFD) group: obese animals fed with HFD (60% of energy as fat, 20% protein, and 20% carbohydrates; PF4215, Mucedola, Milan, Italy) for 16 weeks. The HFD group was used as a control of obesity-related dysfunctions because these animals, consequent to a HFD, develop obesity, hyperglycemia [26,27], hepatic steatosis [28], atherosclerosis [29], and neurodegeneration [30]. (3) HFD-P group: obese animals fed with a HFD supplemented with pistachio (HFD-P) for 16 weeks. 

HFD-P was custom designed and prepared by Mucedola S.R.L (PF4215/C; R&S 34/16). It was obtained by substituting 20% of the caloric intake from HFD with pistachio (180 g/Kg of HFD). The composition of the different diets are provided in Table 1. Mineral and vitamin mix formulas are shown in Tables S1 and S2. Pistachio nuts belong to *Pistacia vera* L. species and were purchased by Pistachio Valle del Platani Association and Pistacchio di Raffadali (Agrigento-AG, Sicily). Previous analysis of the fat content in this Sicilian cultivar highlighted a very high quantity of monounsaturated and polyunsaturated fatty acids (70% oleic acid, 1% palmitoleic acid, and 18% of linoleic fatty acid) [9]. The differences in mono- and polyunsaturated fatty acids between HFD and HFD-P are reported in Table 2.

### 2.3. Reversal Study

Prior to the start of the experiment, mice were randomly assigned to a diet group: either the STD group or HFD group. After 12 weeks on their respective diet, basal glycaemia, triglycerides, and cholesterol plasma concentration were analyzed in each HFD mouse to evaluate HFD-induced dysmetabolism. Any HFD mouse that showed metabolic parameters similar to the lean group was removed from the study. One week following the analysis, mice fed HFD were randomly divided into two sub-groups: one group fed with HFD and the other one fed with HFD added with pistachio for further 16 weeks. 

In both protocols, changes in body weight and food intake were weekly measured and compared among the different groups of animals, as previously described [28]. Blood glucose, triglyceride, and cholesterol concentrations were measured in vivo in mice fasted for 6 h with free access to water, by tail vein puncture using a glucometer (GlucoMen LX meter, Menarini, Florence, Italy) and Biochemistry Analyzer MultiCare-in (Biochemical Systems International-Srl, Arezzo, Italy), respectively. In both protocols, after HFD-P for 16 weeks, mice were sacrificed. Liver and visceral adipose tissue were rapidly dissected and weighed to calculate liver index (index (%) = liver weight (g)/body weight (g) × 100) and fat index (index (%) = visceral adipose tissue weight (g)/body weight (g) × 100), respectively. Liver or visceral adipose tissue part was fixed in 4% neutral formalin solution for histological analysis and another part was used for biomolecular analysis. 

### 2.4. Biochemical Analysis

In euthanized mice, blood was drawn by cardiac puncture and immediately transferred into chilled tubes containing a final concentration of 1 mg/mL ethylenediaminetetraacetic acid (EDTA). Then, the samples were centrifuged at 3000 rpm for 10 min, and the obtained plasma was stored at −80 °C until analysis. aspartate transaminase (AST) and alanine transaminase ALT concentrations were measured using the ILAB 600 Analyzer (Instrumentation Laboratory, Bedford, Massachusetts).

### 2.5. Micro-Computed X-Ray Tomography 

Both in prevention and reversion studies, micro-computed tomography (micro-CT) scans were performed to assess the volumes of the visceral adipose tissue (VAT) and subcutaneous adipose tissue (SAT) depots. 

Four mice from each group were randomly selected and anesthetized with 5% isoflurane. Transverse micro-CT images of the abdomen from L1 to L5 were obtained by the micro-CT scanner Quantum FX µCT (Perkin-Elmer, Hopkinton, MA, USA). Voltage was set at 50 kV and current was set at 200 μA and the images were captured over a 4.5 min interval. Analysis of micro-CT images was conducted with AnalyzePro software (AnalyzeDirect, Overland Park, KS, USA). Visceral and subcutaneous adipose tissue were segmented in the sagittal plane and tissue volumes were expressed relative to body mass [31]. Experimental data from micro-CT were provided by ATeN Center—Università di Palermo.

### 2.6. Histological Analysis

For the microscopic examination of hepatic and adipose tissue morphology, liver and visceral adipose tissue (including epididymal and retroperitoneal adipose tissue) were fixed in 4% buffered formalin for 24 h. Then, the tissues were dehydrated in alcohol and embedded in paraffin. Paraffin histological sections (5 µm thick) were stained with hematoxylin and eosin and observed using an optical microscope (Leica DMLB, Meyer instruments, Houston, Texas) connected to a high-resolution camera (DS-Fi1, Nikon, Florence, Italy). Grading of steatosis was determined by analyzing the morphology and percentage of lipid vesicles in hepatocytes by an experimenter blinded to treatment conditions. The steatosis was defined as absent, light, moderate, or severe when ≤1%, 30%, 30–60%, or ≥60% of the hepatocytes were respectively involved [32]. The size of adipocytes was measured according to the cell diameter [33]. Twenty-thirty adipocytes were measured in different randomly selected optical fields. 

### 2.7. Quantification of Hepatic Lipids

Total liver lipids were extracted using a protocol adapted from Folch et al. [34]. Briefly, the samples were homogenized in ice-cold chloroform:methanol (2:1) solution for 1 min. The homogenate was centrifuged to recover the liquid phase. The solvent was washed with one-quarter of total volume of 0.9% NaCl solution and vortexed vigorously for 30 s. The mixtures were centrifuged at 2000× *g* for 5 min to separate the two phases. The lower phase containing lipids was evaporated under vacuum in a rotary evaporator. The weight difference between the starting empty tube and the tube containing the dried lipids was the lipid amount.

### 2.8. Reverse Transcription Polymerase Chain Reaction (RT-PCR) 

RNA was extracted from liver and visceral adipose tissue using the RNeasy plus Mini Kit (Qiagen, Valencia, CA, USA) according to the manufacturer’s protocol. The extraction from adipose tissue was performed after a preliminary step of lysis using Triazol. Two nanograms of total RNA were used for cDNA synthesis with High Capacity cDNA Reverse Transcription (Applied Biosystems, MA, USA). The target cDNA was amplified using genetic-specific primers, as listed in Table 3. The amplification cycles included denaturation at 95 °C for 10 s, annealing at 60 °C for 15 s, and elongation at 72 °C for 15 s. After 35 cycles, the PCR products were separated by electrophoresis on a 1.8% agarose gel for 45 min at 85 V. The gels were stained with 1 mg/mL ethidium bromide and visualized with ultraviolet (UV) light using E-Gel GelCapture (Thermo Fisher Scientific, Monza, Italy), and the expression levels of the gene targets, normalized to the endogenous reference (β-actin), were analyzed using E-Gel GelQuant Express Analysis Software (Thermo Fisher Scientific, Monza, Italy).

### 2.9. Statistical Analyses

Results are shown as means ± the standard error of the mean (S.E.M.). The letter n indicates the number of animals. Statistical analyses were performed using Prism Version 6.0 Software (Graph Pad Software, Inc., San Diego, CA, USA). The comparison between the groups was performed by ANOVA followed by Bonferroni’s post-test. A *p*-value ≤ 0.05 was considered statistically significant.

## 3. Results

### 3.1. Prevention Study

#### 3.1.1. Effect of Pistachio Consumption on Metabolic Parameters 

**Figure 1:**
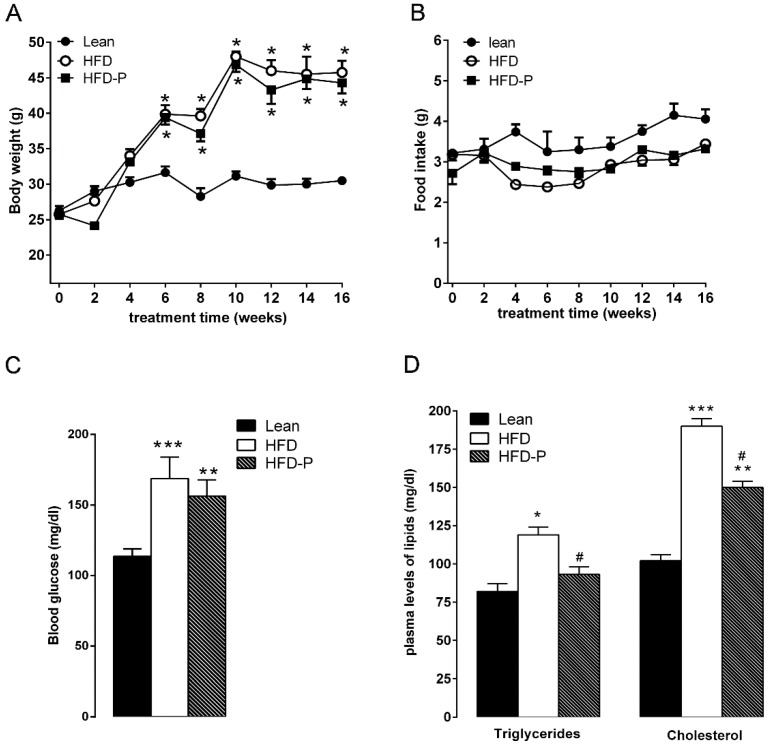
Effects of pistachio consumption on metabolic parameters. Pistachio consumption prevents high-fat diet (HFD)-induced hypertriglyceridemia and hypercholesterolemia. Body weight (**A**), food intake (**B**), fasting glycaemia (**C**), and plasma lipid levels (**D**) in lean, HFD, and HFD-P mice. Data are the means ± S.E.M. (*n* = 8/group). Asterisk denotes significant difference compared with the lean group (* *p* < 0.05; ** *p* < 0.01; *** *p* < 0.001); hash denotes significant difference compared with the HFD group (^#^ *p* < 0.05).

After 16 weeks on diet, HFD mice exhibited a significant increase in body weight in comparison with the STD group. Similarly, mice fed with HFD-P were heavier than the lean group but with a mean body weight similar to HFD animals (Figure 1A). No difference in the daily food intake was observed among the three different groups (Figure 1B). Moreover, HFD mice showed fasting glycaemia, triglyceride, and cholesterol plasma levels higher than the STD group (Figure S1C,D) confirming an impairment of glucose and lipid metabolism [27,35,36]. Pistachio consumption did not prevent HFD-induced hyperglycemia (Figure 1C). On the contrary, triglyceride and cholesterol concentrations were significantly reduced in HFD-P mice in comparison with untreated obese mice, although these values were higher than the STD group (Figure 1D). 

#### 3.1.2. Pistachio Consumption and Liver Steatosis

**Figure 2:**
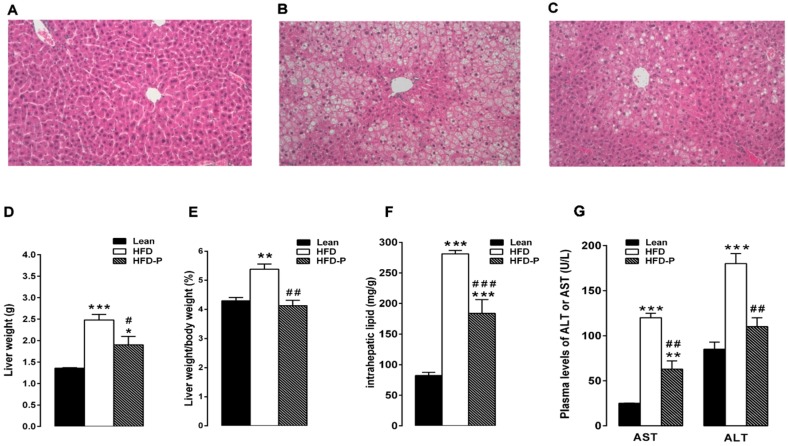
Pistachio consumption and liver steatosis. Pistachio diet prevents hepatic steatosis development in obese mice. Histological cross-sections of liver from lean (**A**), HFD (**B**), and HFD-P mice (**C**). Hematoxylin and eosin stain. Original magnification: ×200. Liver weight (**D**), liver weight/body weight ratio (**E**), intrahepatic lipid content (**F**), and plasma levels of AST and ALT (**G**) in lean, HFD, and HFD-P mice. Data are the means ± S.E.M. (*n* = 8/group). Asterisk denotes significant difference compared with the lean group (* *p* < 0.05; ** *p* < 0.01; *** *p* < 0.001); hash denotes significant difference compared with the HFD group (^#^ *p* < 0.05; ^##^ *p* < 0.01; ^###^ *p* < 0.001).

The liver samples from STD mice showed normal lobular architecture with absence of steatosis (Figure 2A). After 16 weeks of HFD, the obese mouse liver showed deranged structure of hepatic parenchyma with diffuse fatty infiltration corresponding to moderate steatosis (Figure 2B). In agreement with the high accumulation of fat droplets, the hepatic lipid content as well as the absolute and relative (%) liver weight and plasma AST and ALT concentrations were higher in the HFD group than the lean group (Figure 2D–G). Pistachio consumption prevented HFD-induced liver injury; in fact, HFD-P liver showed light steatosis with small lipid inclusion into hepatocytes (Figure 2C). In agreement with the morphological improvements, a significant decrease in the liver fat and weight, liver weight/body weight ratio, and serum aspartate aminotransferase (AST) and alanine aminotransferase (ALT) was observed in HFD-P animals in comparison with the HFD group (Figure 2D–G). 

#### 3.1.3. Pistachio Consumption and HFD-Induced Adipose Tissue Alterations

**Figure 3:**
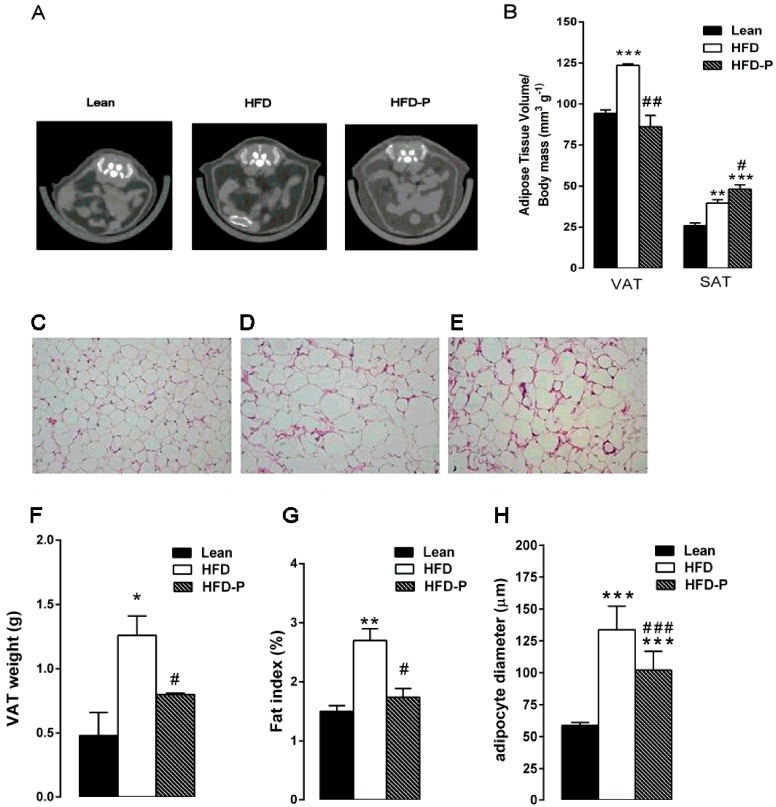
Pistachio consumption and HFD-induced adipose tissue alterations. Pistachio diet prevents visceral fat accumulation. Transverse micro-computed tomography (micro-CT) images of the abdomen (**A**) and visceral adipose tissue (VAT) and subcutaneous adipose tissue (SAT) volume (**B**) in lean, HFD, and HFD-P mice. Histological cross-sections of VAT from lean (**C**), HFD (**D**), and HFD-P mice (**E**). Hematoxylin and eosin stain. Original magnification: ×200. VAT weight (F), VAT weight normalized to body weight (fat index) (**G**), and adipocyte diameter (**H**) in lean, HFD, and HFD-P mice. Data are the means ± S.E.M. (*n* = 8/group). Asterisk denotes significant difference compared with the lean group (* *p* < 0.05; ** *p* < 0.01; *** *p* < 0.001); hash denotes significant difference compared with the HFD group (^#^ *p* < 0.05; ^##^ *p* < 0.01; ^###^ *p* < 0.001).

Micro-computed tomography analysis revealed that the visceral and subcutaneous depots were significantly increased in HFD mice compared to lean animals. Interestingly, HFD-P mice showed a significant reduction in VAT volume/body mass ratio and a significantly increased SAT volume/body mass ratio in comparison with obese animals (Figure 3A–B).

Moreover, histological examination of HFD-VAT revealed a significant increase in adipocyte size, adipose tissue weight, and fat index when compared to the lean group. Once more, HFD-P mice showed significantly reduced adipose tissue weight, fat index, and adipocyte size in comparison with HFD mice, although the values were higher than the STD group (Figure 3C–H).

#### 3.1.4. Effect of Pistachio Consumption on Lipid Metabolism-Related Genes Expression

**Figure 4:**
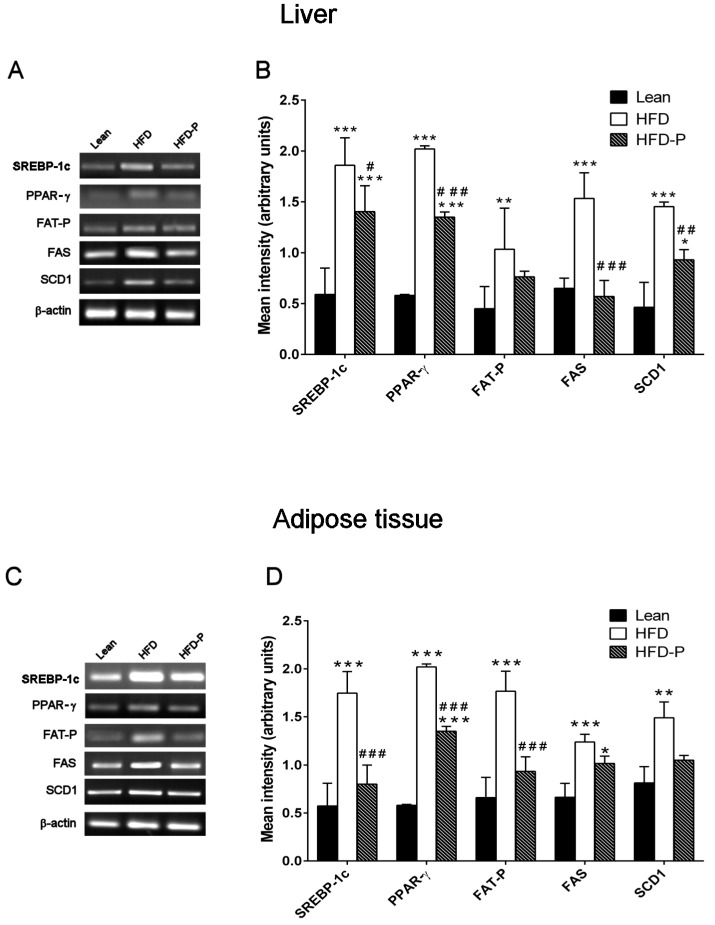
Effect of Pistachio consumption on lipid metabolism-related genes expression. Pistachio intake prevents the impairment of lipid metabolism-related gene expression in liver and adipose tissue. Representative images of the RT-PCR results (left panel) and mRNA levels of *SREBP-1c, PPAR-ɣ, FAT-P, FAS*, and *SCD-1* (right panel) in the livers (**A,B**) and adipose tissues (**C,D**) of lean, HFD, and HFD-P mice (**B**). Data are the means ± S.E.M. (*n* = 8/group). Asterisk denotes significant difference compared with the lean group (* *p* < 0.05; ** *p* < 0.01; *** *p* < 0.001); hash denotes significant difference compared with the HFD group (^#^ *p* < 0.05; ^##^ *p* < 0.01; ^###^ *p* < 0.001).

*SREBP-1c*, *PPAR-γ*, *FAT-P*, *FAS*, and *SCD1* mRNA levels were significantly higher in HFD liver and adipose tissue compared to the gene expression levels observed in lean mice. On the contrary, pistachio-diet significantly normalized *PPAR-γ*, *FAS*, and *SCD1* gene expression changes in liver and *SREBP-1c*, *PPAR-γ*, and *FAT-P* gene expression in adipose tissue, compared with the obese group (Figure 4A–D).

### 3.2. Reversal Study

#### Pistachio Consumption Improves HFD-Induced Lipid Dysmetabolism

The observation that pistachio consumption prevented HFD-induced dyslipidemia as well as fat accumulation in liver and adipose tissue led us to investigate if pistachio consumption is also able to reverse or to improve lipid dysmetabolism. Therefore, after 12 weeks on HFD the mice with ascertained dyslipidemia were further sub-divided into two groups, which received either HFD or HFD-P for 16 additional weeks.

**Figure 5:**
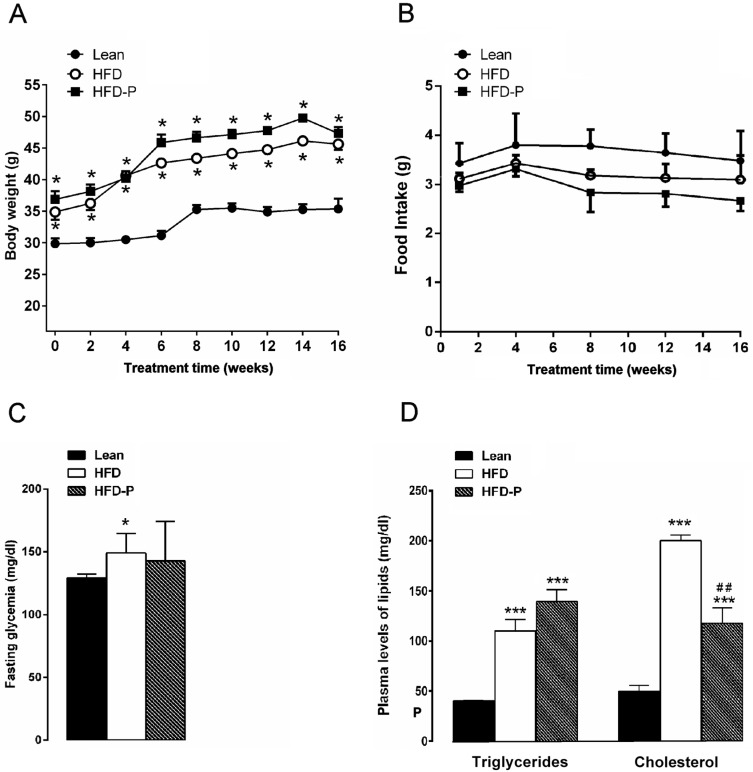
Pistachio consumption effects on body weight, food intake, fasting glycaemia, and plasma lipid levels in mice with ascertained obesity. Pistachio regular intake improves hypercholesterolemia. Body weight (**A**), food intake (**B**), fasting glycaemia (**C**), and plasma lipid levels (**D**) in lean, HFD, and HFD-P mice. Data are the means ± S.E.M. (*n* = 8/group). Asterisk denotes significant difference compared with the lean group (* *p* < 0.05; *** *p* < 0.001); hash denotes significant difference compared with the HFD group (^##^ *p* < 0.01).

No difference in body weight, food intake, and hyperglycemia was observed between HFD and HFD-P mice (Figure 5A–C). Plasma triglyceride levels remained unchanged. However, cholesterol concentration was significantly lower in HFD-P than HFD mice but was significantly higher in respect to the STD mice (Figure 5D).

**Figure 6:**
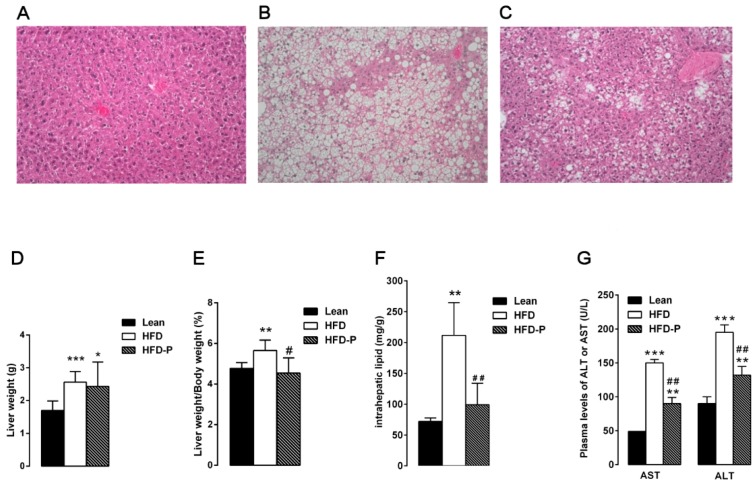
Pistachio consumption exerts **positive** effects on liver steatosis. Histological cross-sections of liver from lean (**A**), HFD (**B**), and HFD-P (**C**) mice belonging to reversal protocol. Hematoxylin and eosin stain. Original magnification: ×200. Liver weight (**D**), ratio of liver weight/body weight (**E**), intrahepatic lipid content (**F**), and plasma levels of AST and ALT (**G**) in lean, HFD, and HFD-P mice belonging to reversal protocol. Data are the means ± S.E.M. (*n* = 8/group). Asterisk denotes significant difference compared with the lean group (* *p* < 0.05; ** *p* < 0.01; *** *p* < 0.001); hash denotes significant difference compared with the HFD group (^#^ *p* < 0.05; ^##^ *p* < 0.01).

When sacrificed, HFD mice showed severe steatosis (Figure 6B), whereas HFD-P mice showed moderate steatosis (Figure 6C). Accordingly, the intrahepatic lipid content as well as liver index and AST and ALT concentration were significantly reduced in HFD-P mice in comparison with HFD mice (Figure 6D–G).

**Figure 7:**
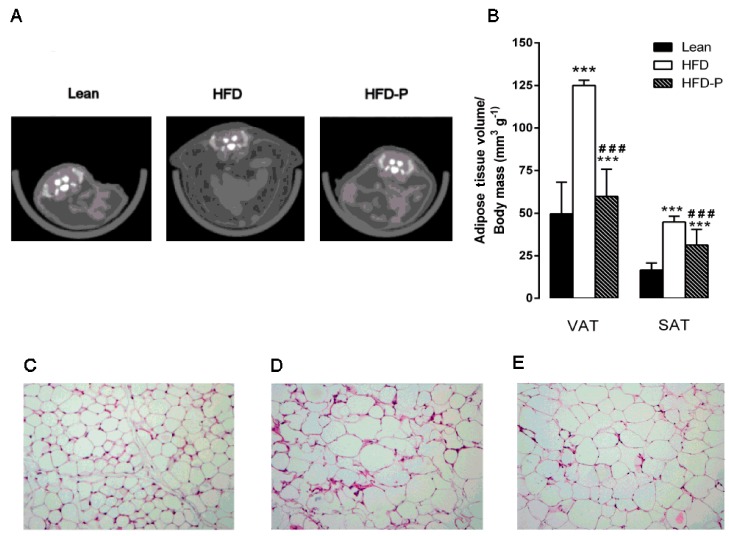

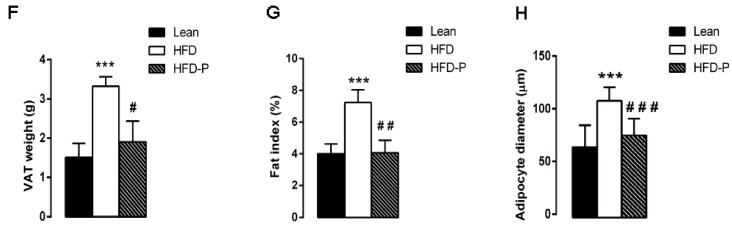
Pistachio consumption reduces visceral fat accumulation. Transverse micro-CT images of the abdomen (**A**) and visceral adipose tissue (VAT) and subcutaneous adipose tissue (SAT) volumes (**B**) in lean, HFD, and HFD-P mice belonging to reversal protocol. Histological cross-sections of VAT from lean (**C**), HFD (**D**), and HFD-P mice (**E**). Hematoxylin and eosin stain. Original magnification: ×200. VAT weight (**F**), VAT weight normalized to body weight (fat index) (**G**), and adipocyte diameter (**H**) in lean, HFD, and HFD-P mice. Data are the means ± S.E.M. (*n* = 8/group). Asterisk denotes significant difference compared with the lean group (*** *p* < 0.001); hash denotes significant difference compared with the HFD group (^#^ *p* < 0.05; ^##^ *p* < 0.01; ^###^ *p* < 0.001).

The analysis of adipose tissue volumes revealed a significant decrease of VAT and SAT in HFD-P mice in comparison with HFD mice. Moreover, adipocyte size, adipose tissue weight, and fat index were significantly reduced in HFD-P mice compared to HFD mice (Figure 7A–H).

**Figure 8:**
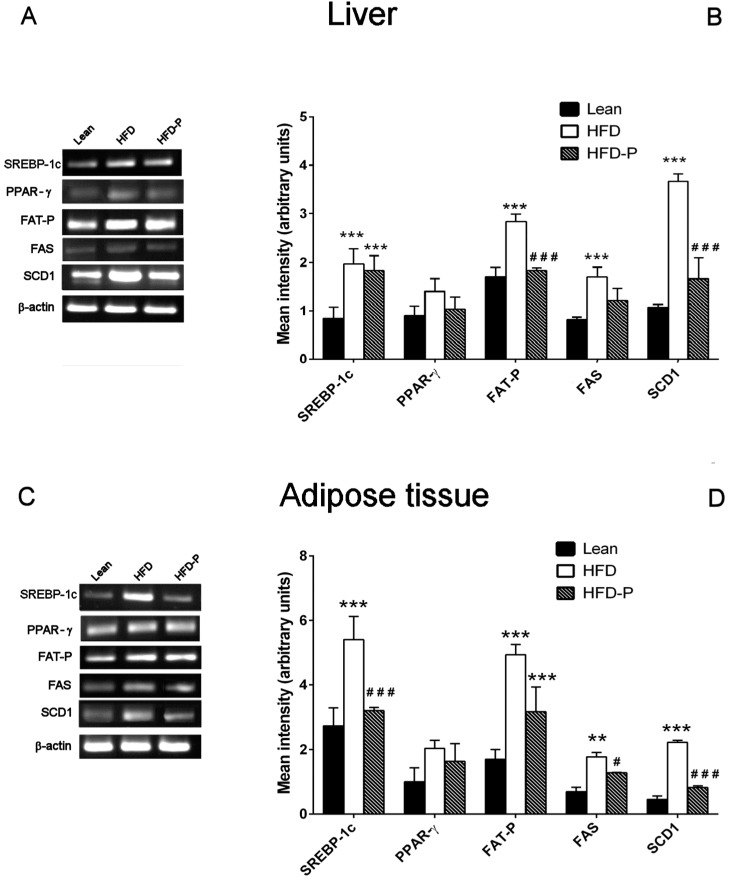
Pistachio consumption reduces the upregulated expression of genes involved in lipid metabolism. Representative images of the RT-PCR results (left panel) and mRNA levels of *SREBP*-*1c*, *PPAR-γ*, *FAT-P*, *FAS*, and *SCD1* (right panel) in the livers (**A–B**) and adipose tissues (**C–D**) of lean, HFD, and HFD-P mice belonging to the reversal protocol. Data are the means ± S.E.M. (*n* = 8/group). Asterisk denotes significant difference compared with the lean group (0.05; ** *p* < 0.01; *** *p* < 0.001); hash denotes significant difference compared with the HFD group (^#^ *p* < 0.05; ^###^ *p* < 0.001).

The analysis of gene expression revealed that HFD-P significantly reduced *FAT-P* and SCD1 expression in liver and *SREBP*-*1c*, *FAS*, and *SCD1* expression in adipose tissue (Figure 8 A–D).

## 4. Discussion

The present study demonstrates that regular pistachio intake is able to prevent and to improve obesity-related metabolic dysfunctions such as dyslipidemia, hepatic steatosis, and adipose tissue alterations in HFD obese mice. These beneficial effects could be due to the positive modulation of lipid metabolizing gene expression.

Compared to other nuts, pistachios represent a potentially functional food in preventing obesity-related metabolic dysfunctions. In fact, they are a rich source of unsaturated fatty acids and antioxidant substances, such as γ-tocopherol, β-carotene, lutein, selenium, flavonoids, and phytosterols [9,37,38].

Indeed, in vitro and in vivo studies have highlighted healthy properties of pistachios, which could be attributed to the content of antioxidant substances (proanthocyanidins, epicatechin, isoquercetin, γ-tocopherol) [16,39,40,41,42]. Moreover, several studies on humans have provided evidence of the beneficial effects of pistachio on cardiovascular risk markers, including blood lipid levels [20,21,43], blood pressure [44], oxidative stress [23] endothelial dysfunctions [43], and glucose dysmetabolism [19,45].

Our goal was to evaluate the ability of pistachio intake to prevent (from obesity induction starting) or revert (after obesity achievement) metabolic obesity-related disorders in mice.

In our experimental conditions, pistachio consumption did not modify the HFD-induced body weight increase or the food intake, in accordance with epidemiological studies showing that regular intake of pistachios is not related to weight gain [19,23,24,43,46].

Moreover, pistachio intake failed to prevent HFD-induced hyperglycemia. Previous studies have reported contradictory results about the effects of pistachio consumption on glucose metabolism, depending on the subjects examined (healthy or affected by MetS) or on the period in which pistachios are consumed [19,43,46]. Indeed, only a long-term consumption appears to have beneficial effects on the obesity-related glucose dysmetabolism [46], although in our experiments, 16 weeks of HFD-P did not improve the HFD-induced hyperglycemia.

In the present study, pistachio intake was able to prevent and to improve the plasma dyslipidemia and the lipid accumulation in liver and adipose tissue, providing evidence for lipid lowering properties of pistachios. These findings are in agreement with previous results obtained in animals [42,47] and humans [20,21,22,23,43,48,49].

Different mechanisms could be responsible for the hypolipidemic effects of pistachio consumption. They could be caused by the high content of MUFA and PUFA because it is well known that the consumption of unsaturated fatty acids reduces plasma LDL and triglyceride levels [50]. In fact, as reported in Table 2, there are differences in fatty acids composition between the HFD and HFD-P. Alternatively, or in addition, they could be due to the high levels of phytosterols. In fact, a diet-supplemented with phytosterols causes inhibition of cholesterol absorption in the gastrointestinal tract [51].

Although a high intake of nuts (in particular, walnuts) can improve liver function in patients with hepatic steatosis and be positively linked to a lower risk of NAFLD developing [52,53], there are no data available about regular pistachio intake and hepatic function. Our results show for the first time that pistachio consumption exerts preventive and improving effects on hepatic steatosis, fat liver accumulation, and hepatic functions. In fact, liver index and ALT and AST plasma levels were significantly lower in HFD-P mice. 

It is well accepted that dietary fat composition influences the expression of the **genes** controlling hepatic lipid metabolism [54,55]. Accordingly, we investigated if the beneficial effects of pistachio consumption on hepatic steatosis could be due to changes in the expression of the transcription factors *PPAR-γ* and *SREBP-1c* with their target genes *FAS* and *SCD1*, which are the principal regulators of fatty acid synthesis, and *FAT-P*, which is involved in fatty acid uptake from the extracellular milieu [56].

Our RT-PCR analysis revealed that the gene expression of *PPAR-γ*, *SREBP-1c*, *FAS*, *SCD1*, and *FAT-P* was upregulated in the liver of HFD mice compared to STD mice, confirming that HFD induced impairment of the lipid metabolizing gene expression that is involved in steatosis [4,6,57,58,59]. However, HFD-P was able to prevent *PPAR-γ*, *FAS*, and *SCD1* upregulation and to ameliorate severe steatosis in the liver of obese mice, suggesting that pistachio consumption exerts hypolipidemic effects by preventing hepatic de novo lipogenesis impairment and by reducing fatty acid uptake.

Our results represent the first experimental data showing the ability of pistachio consumption to modulate lipid gene expression. On the other hand, several bioactive plant components [6,57,60] as well as functional foods [60,61,62] are able to counteract hepatic steatosis by positively modulating the expression of genes linked to lipid metabolism.

Moreover, our results suggest that a pistachios-based diet is able to prevent fat mass accumulation because VAT, fat-mass, fat-index, and adipocyte diameter were significantly reduced in HFD-P mice in comparison with HFD mice. Interestingly, SAT volume was increased in HFD-P mice, suggesting that pistachio consumption could be responsible for an adipose tissue redistribution linked to a healthier profile. In fact, unlike VAT, which is strictly associated with cardiometabolic risk [63,64], SAT shows some protective metabolic features [65,66,67]. The role of functional foods or natural extracts on the specific regional adiposity had never been clearly established. In terms of previous research, just a multi-ethnic study revealed a link between a healthy dietary pattern (including nuts) and lower visceral fat [65]. As such, our results are the first evidence regarding the ability of a functional food to influence adipose tissue redistribution.

In the reversion protocol, fat-mass, fat-index, adipocyte diameter, and VAT and SAT volumes were significantly decreased in HFD-P mice compared to HFD mice, suggesting that regular pistachio intake is able to reduce fat mass accumulation even when a state of obesity was established. It is interesting to note that pistachio consumption was more efficacious in reducing VAT volume than SAT, confirming that pistachio consumption is able to counteract the VAT noxious fat depot. 

Lastly, our results also provide evidence for a modulator role of regular pistachio intake on the expression of genes involved in lipid metabolism in adipose tissue. In fact, the addition of pistachio to the diet significantly prevented HFD-induced upregulation of *SREBP-1c*, *PPAR-γ*, and *FAT-P* and reduced *SREBP-1c*, *FAS*, and *SCD1* over-expression in the reversion study, suggesting a decrease in de novo lipid synthesis and lipid uptake in the adipose tissue.

## 5. Conclusions

In conclusion, our results suggest that pistachio may act as an effective functional food, which may be useful to prevent as well as to ameliorate obesity-related metabolic disorders such as hyperlipidemia, hepatic steatosis, and adipose tissue fat accumulation. Regular pistachio intake could exert beneficial effects on lipid metabolism by reducing the expression of lipid metabolism-related genes in liver and adipose tissue. 

## Figures and Tables

**Table 1:** Composition and energy densities of STD, HFD, and HFD-P.

Ingredient (g/kg)	STD	HFD	HFD-P
Acid Casein 741	200	265.00	210.00
L-Cystine	2.8	4	4
Maltodextrine-0032	33.2	160	125.5
Sucrose	300	90	100
Cellulose (Arbocel)	50	65.5	50
Soybean Oil	25	30	30
Lard	19	220	135
Vitamin mix AIN-93-VX-PF2439	10	21	21
Mineral mix AIN-93G-MX-PF2348	45	48	48
Choline Bitartrate	1.9	3	3
Calcium Phosphate Dibasic	13	3.4	3.4
Pistachio	-	-	180
Total Energy, Kcal/g	3.5	6	6
Protein, %	20	20	20
Carbohydrate, %	70	20	20
Fat, %	10	60	60

Abbreviations are: STD, standard diet; HFD, high fat diet; HFD-P, HFD supplemented with pistachio. This study used 4RF25, PF4051/D, and PF4215/C-R&S34/16 diets (Mucedola S.R.L.) as STD, HFD, and HFD-P, respectively. Composition of these diets is from the Mucedola website. The mineral and vitamin mix formulas are shown in Tables S1 and S2, respectively.

**Table 2:** Fatty acids composition in HFD and HFD-P.

Fatty Acids (g/kg)	HFD	HFD-P
total saturated	92.5	71.12
total monounsaturated	117.5	136.65
total polyunsaturated	40	42.23

Abbreviations are: HFD, high fat diet; HFD-P, HFD supplemented with pistachio.

**Table 3:** Oligonucleotide sequence of primers for RT-PCR.

Gene	Forward Primer	Reverse Primer	Size (bp)
*FAS*	5′-TACTTTGTGGCCTTCTCCTCTGTAA-3′	5′-CTTCCACACCCATGAGCGAGTCCAGGCCGA-3′	445
*SCD1*	5′-GCCAGACCGGGCTGAACACC-3′	5′-GGCCTCCCAAGTGCAGCAGG-3′	397
*SREBP-1c*	5′-GGAGACATCGCAAACAAGC-3′	5′-GGTAGACAACAGCCGCATC-3′	273
*PPAR-ɣ*	5′-GGGCTGAGGAGAAGTCACAC-3′	5′-TCAGTGGTTCACCGCTTCTT-3′	142
*FAT-P*	5′-CGCCGATGTGCTCTATGACT-3′	5′-ACACAGTCATCCCAGAAGCG-3′	138
*β-actin*	5′-GGATCCCCGCCCTAGGCACCAGGGT-3′	5′-GGAATTCGGCTGGGGTGTTGAAGGTCTCAAA-3′	289

## Supplementary Materials

The following are available online at http://www.mdpi.com/2072-6643/10/12/1857/s1, Table S1: Composition of mineral mix in STD, HFD, and HFD-P. Table S2: Composition of vitamin mix in STD, HFD, and HFD-P.
