# Supplementary material for: Pistachio Consumption Prevents and Improves Lipid Dysmetabolism by Reducing the Lipid Metabolizing Gene Expression in Diet-Induced Obese Mice
Source: Nutrients. 2018 Dec 1;10(12):1857. doi: 10.3390/nu10121857 (PMC6316241; doi:10.3390/nu10121857)
Supplement: Supplementary file 1 [file nutrients-10-01857-s001.pdf]

TableS1. Composition of mineral mix in STD, HFD and HFD-P.

PMIX AIN-93G-MX	
**Ingredient (mg/kg)**	**4800 g/100 kg**
**Calcium carbonate**	6854,40
**Potassium phosphate monobasic**	2700,10
**Potassium citrate tribasic monohydrate**	1223,08
**Sodium chloride**	1395,94
**Magnesium oxide**	703,34
**Potassium sulfate**	1004,32
**Chromium K sulfate**	1,37
**Cupric carbonate**	8,26
**Sodium fluoride**	1,38
**Potassium iodate**	0,28
**Ferric citrate**	55,27
**Manganese carbonate**	14,45
**Ammonium molybdate**	0,21
**Basic nickel carbonate**	0,15
**Lithium chloride**	0,14
**Boric acid**	0,68
**Ammonium metavanadate**	0,14
**Sodium metasilicate**	6,88
**Zinc carbonate**	45,94
**Sodium selenite**	0,21

TableS2. Composition of vitamin mix in STD, HFD and HFD-P.

PMIX AIN-93G-VX	
**Ingredient (mg/kg)**	**2100 g/100 kg**
**Vit. K1 phylloquinone 97-103%**	1,58 mg/Kg
**Nicotinic acid 99,5-100,5%**	63,0 mg/Kg
**Calcium pantothenate 98-101%**	33,6 mg/Kg
**Vit. A palmitate 250**	8400 I.U./Kg
**Biotin 97,5-100,5%**	0,42 mg/Kg
**Piridoxine 99-101%**	14,70 mg/Kg
**Riboflavin 97-103%**	12,60 mg/Kg
**Thiamine 98,5-101%**	12,60 mg/Kg
**Vit. D3 Cholecalciferol, 500**	2100 I.U./Kg
**Cyanocobalamin > 0,1%**	0,053 mg/Kg
**Folic Acid 96-102%**	4,16 mg/Kg
**α-tocopheryl acetate, 500 IU/g**	157,50 mg/Kg
